# Supplementary material for: Clinical and Transcriptomic Characterization of Metastatic Hormone-Sensitive Prostate Cancer Patients with Low PTEN Expression
Source: Int J Mol Sci. 2025 Jun 28;26(13):6244. doi: 10.3390/ijms26136244 (PMC12249740; doi:10.3390/ijms26136244)

Supplementary Figures

Clinical and Transcriptomic Characterization of Metastatic Hormone-Sensitive Prostate Cancer Patients with Low *PTEN* Expression

Marta Garcia de Herreros*, Natalia Jiménez*, Joan Padrosa*, Caterina Aversa, Laura Ferrer-Mileo, Samuel García-Esteve, Leonardo Rodríguez-Carunchio, Isabel Trias, Laia Fernández-Mañas, Mercedes Marín-Aguilera, Mariana Altamirano, Manuel Mazariegos, Albert Font, Alejo Rodriguez-Vida, Miguel Ángel Climent, Sara Cros, Isabel Chirivella, Mariona Figols, Núria Sala-González, Vicenç Ruiz de Porras, Juan Carlos Pardo, Aleix Prat, Òscar Reig[Ŧ] & Begoña Mellado[Ŧ]

*Equally contributed to this work

[Ŧ]Corresponding authors

page

Figure S1 **2**

Figure S2 **3**

Figure S3 **4**

Figure S4 **5**

Figure S5 **6**

Figure S1. Clinical outcomes according to *PTEN* expression status in the ADT cohort. Kaplan–Meier curves representing CRPC-free survival (CRPC-FS) (**A**) and overall survival (OS) (**B**) according to *PTEN* expression (nCounter) in the ADT cohort; Forest plots representing the univariate (**C-D**) and multivariate (**E-F**) analysis for CRPC-FS and OS in the ADT cohort. ADT: androgen deprivation therapy; CI: confidence interval; m: median months; PSA: Prostate-specific antigen. Significant *p* values (p<0.05) are bold indicated.

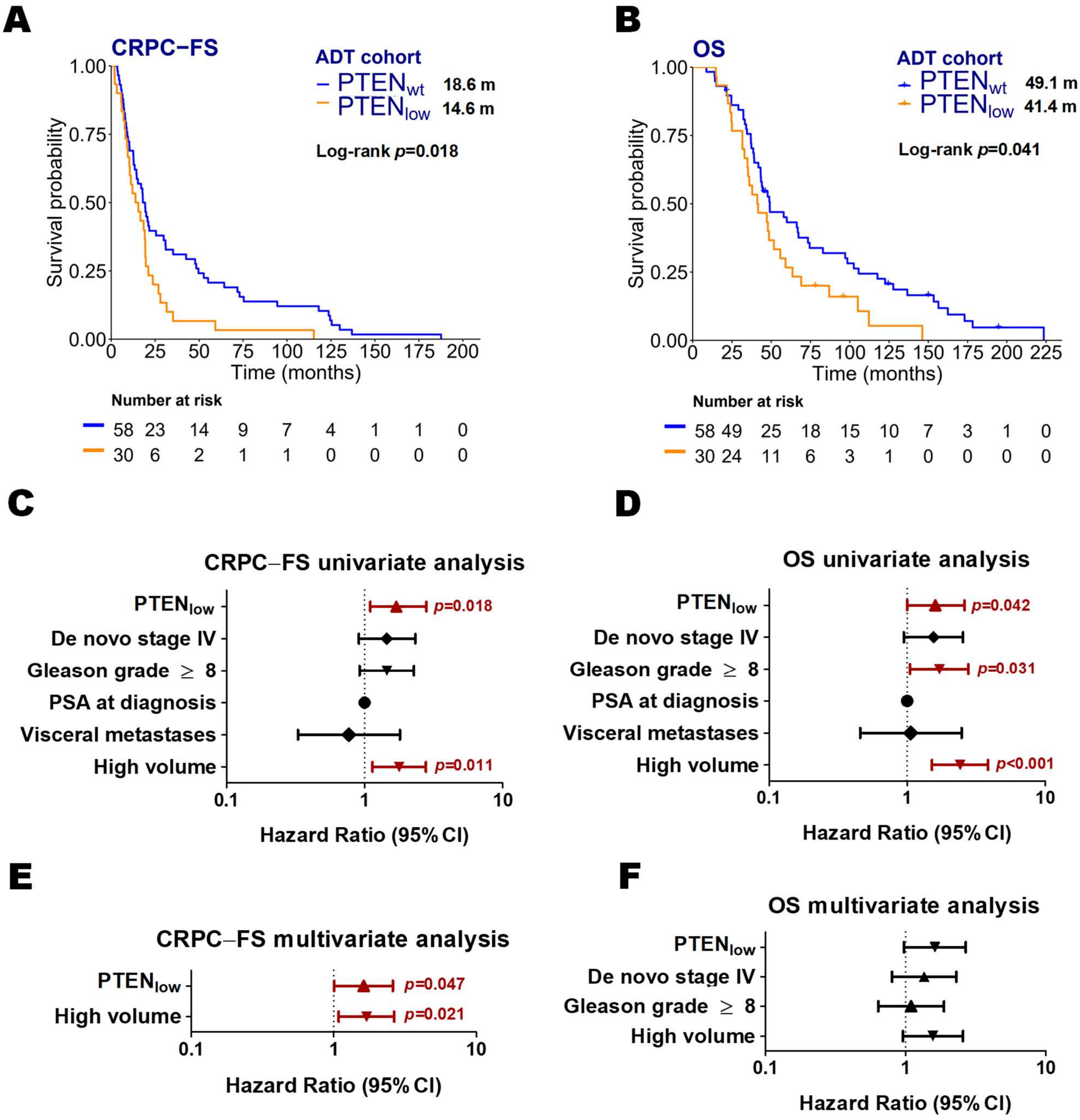

Figure S2. Clinical outcomes according to *PTEN* expression status in the ADT+D cohort. Kaplan–Meier curves representing CRPC-free survival (CRPC-FS) (**A**) and overall survival (OS) (**B**) according to *PTEN* expression (nCounter) in the ADT+D cohort; Forest plots representing the univariate (**C-D**) and multivariate (**E-F**) analysis for CRPC-FS and OS in the ADT+D cohort. ADT: androgen deprivation therapy; CI: confidence interval; D: docetaxel; LDH: lactate dehydrogenase; m: median months. Significant *p* values ($p<0.05$) are bold indicated.

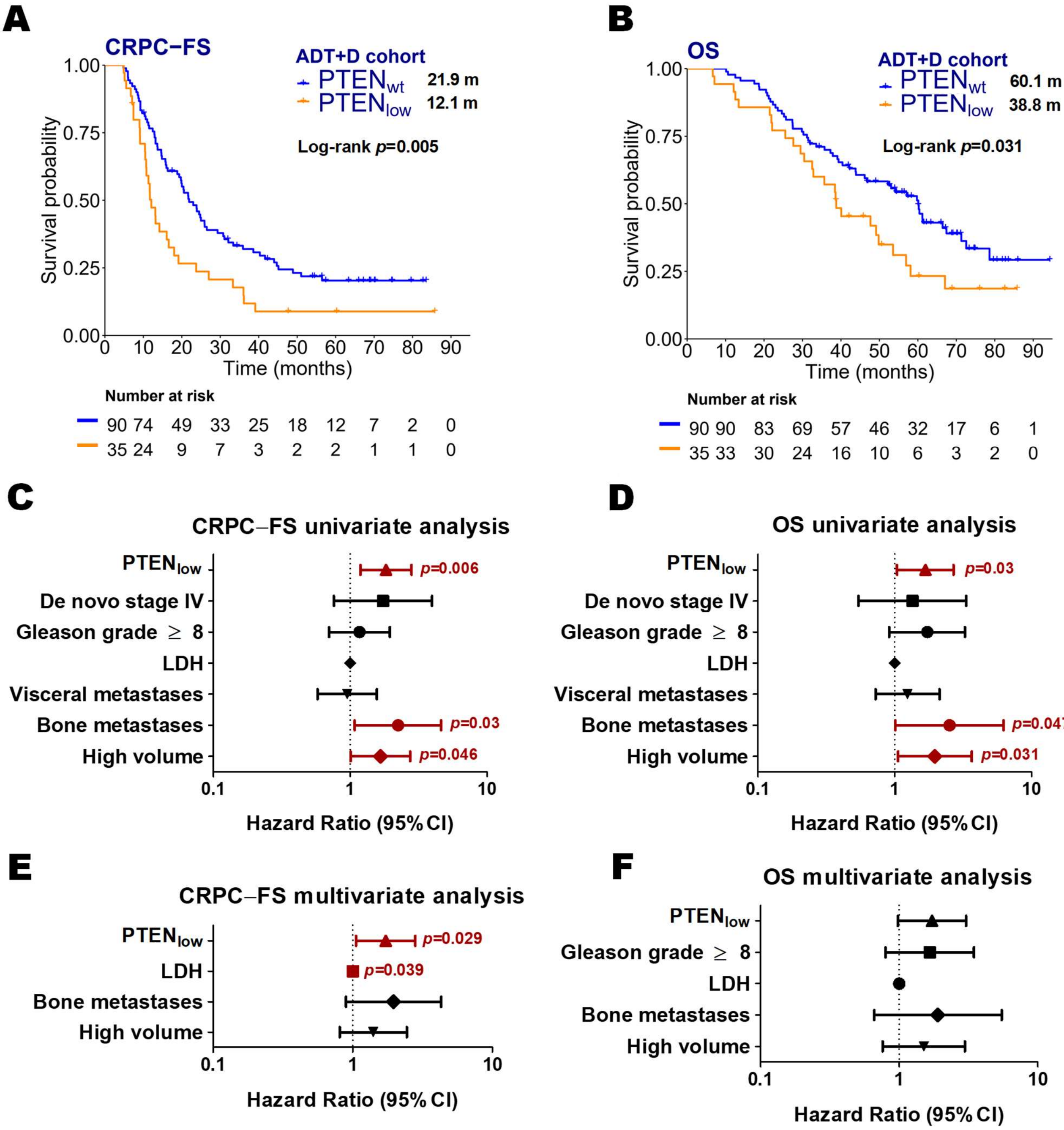

Figure S3. Clinical outcomes according to *PTEN* expression status in the ADT+ARSI cohort. Kaplan–Meier curves representing CRPC-free survival (CRPC-FS) (**A**) and overall survival (OS) (**B**) according to *PTEN* expression (nCounter) in the ADT+ARSI cohort; Forest plots representing the univariate (**C-D**) and multivariates (**E-F**) analysis for CRPC-FS and OS in the ADT+ARSI cohort. ADT: androgen deprivation therapy; ARSI: androgen receptor signalling inhibitors; CI: confidence interval; LDH: lactate dehydrogenase; m: median months; NR: not reached. Significant *p* values ($p<0.05$) are bold indicated.

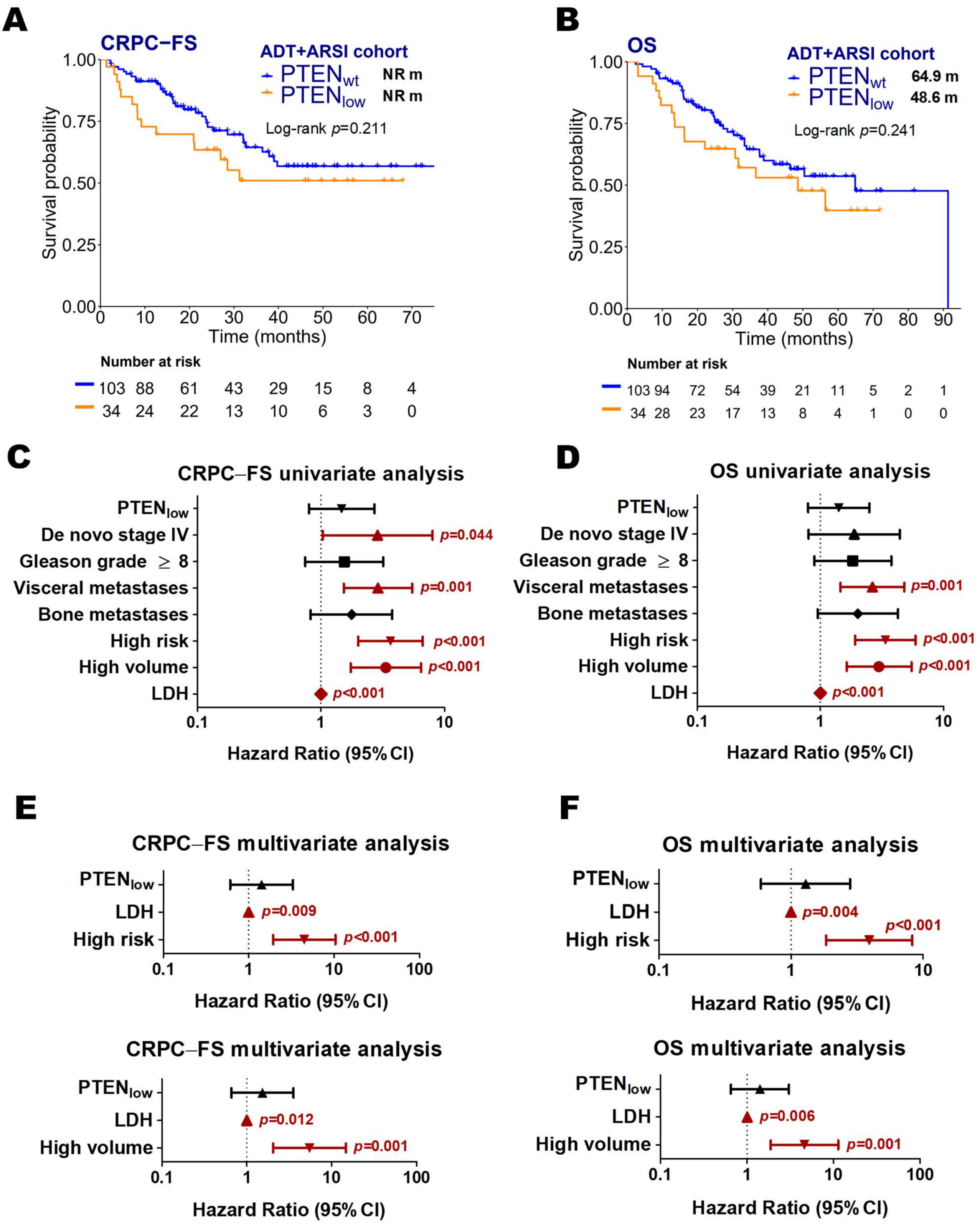

Figure S4. Clinical outcomes according to *PTEN* and *EZH2* expression. Kaplan–Meier curves representing CRPC-free survival (CRPC-FS) (**A**) and overall survival (OS) (**B**) according to *PTEN* and *EZH2* expression (nCounter) in in all patients. m: median months.

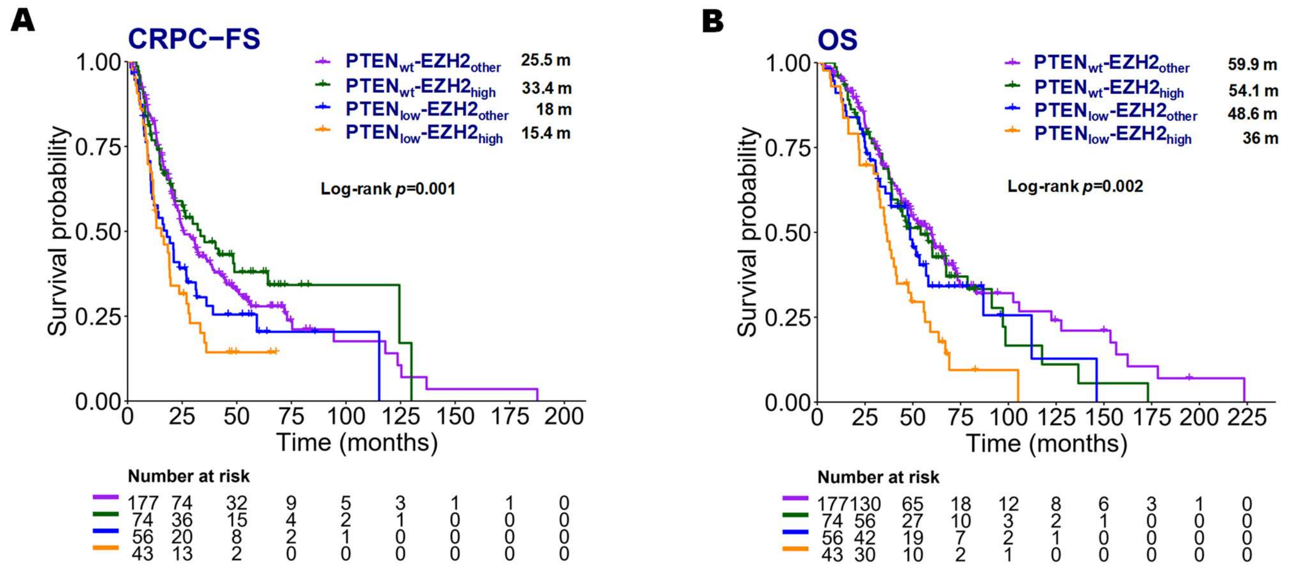

Figure S5. Hallmark pathways associated to PTEN-low related signature. A) Correlation dotplot between the GSVA score of the PTEN-low related signature in the in the training cohort (microarray data from CHAARTED trial) and the GSVA scores of the Hallmark pathways from MSigDB. Triangles represent significant Spearman correlations; **B)** Gene set enrichment analysis of dysregulated Hallmark pathways associated with the PTEN-low related signature in the validation RNA-Seq cohort (*N*=60). Triangles represent significantly enriched pathways (FDR<0.05).

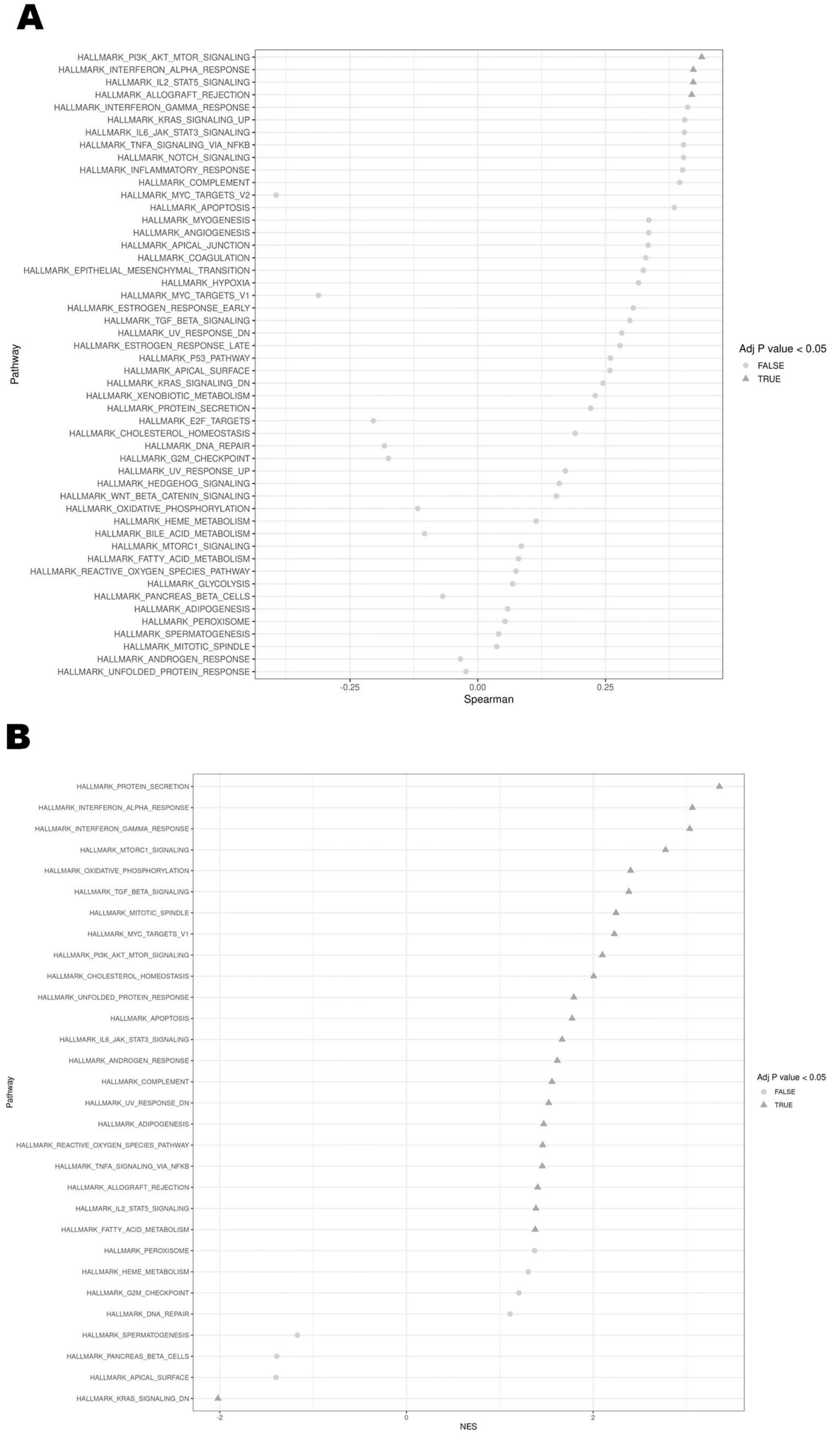

Supplement: Supplementary file 1 [file ijms-26-06244-s001.zip › Supplementary Figures_IJMS_Mellado.pdf]
