# Supplementary material for: Clinical and Transcriptomic Characterization of Metastatic Hormone-Sensitive Prostate Cancer Patients with Low PTEN Expression
Source: Int J Mol Sci. 2025 Jun 28;26(13):6244. doi: 10.3390/ijms26136244 (PMC12249740; doi:10.3390/ijms26136244)
Supplement: Supplementary file 1 [file ijms-26-06244-s001.zip › Supplementary Tables_IJMS_Mellado.pdf]

Supplementary Tables

Clinical and Transcriptomic Characterization of Metastatic Hormone-Sensitive Prostate Cancer Patients with Low *PTEN* Expression

Marta Garcia de Herreros*, Natalia Jiménez*, Joan Padrosa*, Caterina Aversa, Laura Ferrer-Mileo, Samuel García-Esteve, Leonardo Rodríguez-Carunchio, Isabel Trias, Laia Fernández-Mañas, Mercedes Marín-Aguilera, Mariana Altamirano, Manuel Mazariegos, Albert Font, Alejo Rodriguez-Vida, Miguel Ángel Climent, Sara Cros, Isabel Chirivella, Mariona Figols, Núria Sala-González, Vicenç Ruiz de Porras, Juan Carlos Pardo, Aleix Prat, Òscar Reig[Ŧ] & Begoña Mellado[Ŧ]

*Equally contributed to this work

[Ŧ]Corresponding authors

	page
**Table S1**	**2**
**Table S2**	**3**
**Table S3**	**4**
**Table S4**	**5**
**Table S5**	**6**
**Table S6**	**8**
**Table S7**	**11**
**Table S8**	**14**

Table S1. Characteristics of patients from ADT, ADT+D and ADT+ARSI cohorts.

	ADT cohort	ADT+D cohort	ADT+ARSI cohort	*p*-value
Patients, *N* (%)	93 (26.2)	125 (35.2)	137 (38.6)	
Age (years)				
Median (range)	66.1 (51 - 84.61)	66.6 (46.3 – 83.4)	71.8 (50.1 – 92.8)	**<0.001**
PSA at diagnosis (ng/ml)				
Median (range)	24 (0.02 – 6014)	82 (1.8 – 7448)	30 (1.8 – 4600)	**0.001**
ECOG performance status score, *N* (%)				
0	39 (41.9)	54 (43.2)	42 (30.7)	**0.022**
1 or 2	45 (48.4)	69 (55.2)	82 (59.9)	
NA	9 (9.7)	2 (1.6)	13 (9.5)	
Stage at diagnosis, *N* (%)				
<IV	33 (35.5)	9 (7.2)	28 (20.4)	**<0.001**
IV	49 (52.7)	116 (92.8)	106 (77.4)	
NA	11 (11.8)	-	3 (2.2)	
Gleason sum at diagnosis, *N* (%)				
≤7	31 (33.3)	22 (17.6)	31 (22.6)	**0.011**
≥8	56 (60.2)	102 (81.6)	103 (75.2)	
NA	6 (6.5)	1 (0.8)	3 (2.2)	
Presence of visceral metastases, *N* (%)				
Yes	8 (8.6)	25 (20)	22 (16.1)	0.091
No	81 (87.1)	100 (80)	115 (83.9)	
NA	4 (4.3)	-	-	
Disease volume, *N* (%)				
High	51 (54.8)	97 (77.6)	73 (53.3)	**<0.001**
Low	38 (40.9)	27 (21.6)	65 (46.7)	
NA	4 (4.3)	1 (0.8)	-	

ADT: Androgen deprivation therapy; ARSI: Androgen receptor signalling inhibitor; CI: confidence interval; CRPC: Castration-resistant prostate cancer; D: Docetaxel; ECOG: Eastern Cooperative Oncology Group; *N*: number of cases; NA: not available; PSA: Prostate-specific antigen. *p*-value is based on Chi-square test and Kruskal Wallis test for categorical and continuous variables, respectively. Significant *p*-values ($p<0.05$) are bold indicated.

Table S2. Clinical outcomes of patients from ADT, ADT+D and ADT+ARSI cohorts.

	ADT cohort	ADT+D cohort	ADT+ARSI cohort
Patients, *N* (%)	93 (26.2)*	125 (35.2)	137 (38.6)
Follow-up (months)<br>Median (range)	45.8 (8.7 – 223.5)	46.7 (6.7 – 94.3)	30.7 (2.4 – 91.3)
Time to CRPC (months)<br>Median (CI 95%)	17.8 (13.4 – 19.7)	19.3 (14.8 – 23.8)	NR (37.9 – NR)
Overall survival<br>Median (CI 95%)	47.7 (41.1 – 59.1)	53 (42.2 – 60.4)	56.4 (38.8 – NR)

ADT: Androgen deprivation therapy; ARSI: Androgen receptor signalling inhibitor; CI: confidence interval; CRPC: Castration-resistant prostate cancer; D: Docetaxel; *N*: number of cases; NR: not reached. *Five patients were excluded from survival analysis due to lack of complete follow-up data.

Table S3. Characteristics of patients from ADT, ADT+D and ADT+ARSI cohorts segregated according to *PTEN* expression by nCounter.

COHORT	ADT cohort*			ADT+D cohort			ADT+ARSI cohort		
PTEN status	PTEN$_{low}$	PTEN$_{wt}$	*p*-value	PTEN$_{low}$	PTEN$_{wt}$	*p*-value	PTEN$_{low}$	PTEN$_{wt}$	*p*-value
Patients, *N* (%)	31 (33.3)	62 (66.7)		35 (28)	90 (72)		34 (24.8)	103 (75.2)	
Age (years)<br>Median (range)	68.1<br>(51 – 84.6)	65.3<br>(53.2 – 81.2)	0.946	63.7<br>(47.7 – 83.4)	67<br>(46.3 – 81.8)	0.566	72.3<br>(54.9 – 86.5)	71.8<br>(50.1 – 92.8)	0.75
PSA at diagnosis (ng/ml)<br>Median (range)	25<br>(3.2 – 4860)	23<br>(0.02 – 6014)	0.547	79.3<br>(1.8 – 3190)	84.7<br>(5.2 – 7448)	0.96	13.5<br>(3.1 – 2187.5)	35.1<br>(1.8 – 4600)	0.093
ECOG performance status score, *N* (%)<br>0<br>1 or 2<br>NA	12 (38.7)<br>16 (51.6)<br>3 (9.7)	27 (43.5)<br>29 (46.8)<br>6 (9.7)	0.817	12 (34.3)<br>23 (65.7)<br>-	42 (46.7)<br>46 (51.1)<br>2 (2.2)	0.227	11 (32.4)<br>22 (64.7)<br>1 (2.9)	31 (46.7)<br>60 (51.1)<br>12 (11.7)	0.761
Stage at diagnosis, *N* (%)<br><IV<br>IV<br>NA	11 (35.5)<br>17 (54.8)<br>3 (9.7)	22 (35.5)<br>32 (51.6)<br>8 (12.9)	1	1 (2.9)<br>34 (97.1)<br>-	8 (8.9)<br>82 (91.1)<br>-	0.443	4 (11.8)<br>29 (85.3)<br>1 (2.9)	24 (23.3)<br>77 (74.8)<br>2 (1.9)	0.218
Gleason sum at diagnosis, *N* (%)<br>≤7<br>≥8<br>NA	7 (22.6)<br>22 (71)<br>2 (6.5)	24 (38.7)<br>34 (58.8)<br>4 (6.5)	0.155	8 (22.8)<br>27 (77.1)<br>-	14 (15.6)<br>75 (83.3)<br>1 (1.1)	0.434	5 (14.7)<br>27 (79.4)<br>2 (5.9)	26 (25.2)<br>76 (73.8)<br>1 (1)	0.338
Presence of visceral metastases, *N* (%)<br>Yes<br>No<br>NA	1 (3.2)<br>28 (90.3)<br>2 (6.5)	7 (11.3)<br>53 (85.5)<br>2 (3.2)	0.265	9 (25.7)<br>26 (74.3)<br>-	16 (17.8)<br>74 (82.2)<br>-	0.328	10 (29.4)<br>24 (70.6)<br>-	12 (11.7)<br>91 (88.3)<br>-	**0.028**
Disease volume, *N* (%)<br>High<br>Low<br>NA	16 (51.6)<br>13 (41.9)<br>2 (6.5)	35 (56.5)<br>25 (40.3)<br>2 (3.2)	0.88	30 (85.7)<br>4 (11.4)<br>1 (2.9)	67 (74.4)<br>23 (25.6)	0.142	18 (52.9)<br>16 (47.1)	55 (53.4)<br>48 (46.6)	1

ADT: Androgen deprivation therapy; ARSI: Androgen receptor signalling inhibitor; D: Docetaxel; ECOG: Eastern Cooperative Oncology Group; *N*: number of cases; NA: not available; PSA: Prostate-specific antigen. *p*-value is based on Fisher exact test and Wilcoxon Mann–Whitney test for categorical and continuous variables, respectively. *Five patients were excluded from survival analysis due to lack of complete follow-up data. Significant *p*-values ($p<0.05$) are bold indicated.

Table S4. Characteristics of patients with RNA-Seq data (ADT+D treatment) and these patients segregated according to *PTEN* expression by nCounter.

	Patients with RNA-seq data	$PTEN_{low}$	$PTEN_{wt}$	*p*-value
Patients, *N* (%)	60	15 (25)	45 (75)	
Age (years)				
Median (range)	67.2 (49.4 – 83.4)	65.1 (53.6 – 83.4)	67.7 (49.4 – 81.8)	0.946
PSA at diagnosis (ng/ml)				
Median (range)	219 (3 – 7448)	64.8 (3 – 3190)	288 (6.6 – 7448)	0.457
ECOG performance status score, *N* (%)				
0	27 (45)	7 (46.7)	20 (44.4)	0.565
1 or 2	31 (51.7)	8 (53.3)	23 (51.1)	
NA	2 (3.3)	-	2 (4.4)	
Stage at diagnosis, *N* (%)				
<IV	0 (0)	0 (0)	0 (0)	-
IV	60 (100)	15 (100)	45 (100)	
Gleason sum at diagnosis, *N* (%)				
≤7	9 (15)	5 (33.3)	4 (8.9)	**0.036**
≥8	51 (85)	10 (66.7)	41 (91.1)	
Presence of visceral metastases, *N* (%)				
Yes	9 (15)	4 (26.7)	5 (11.1)	0.208
No	51 (85)	11 (73.3)	40 (88.9)	
Disease volume, *N* (%)				
High	48 (80)	13 (86.7)	35 (77.8)	0.712
Low	12 (20)	2 (13.3)	10 (22.2)	

ADT: Androgen deprivation therapy; D: Docetaxel; ECOG: Eastern Cooperative Oncology Group; *N*: number of cases; NA: not available; PSA: Prostate-specific antigen. *p*-value is based on Fisher exact test and Wilcoxon Mann–Whitney test for categorical and continuous variables, respectively. Significant *p*-values ($p<0.05$) are bold indicated.

Table S5: Differentially expressed genes between $PTEN_{low}$ and $PTEN_{wt}$ tumors.

ENSEMBLE ID	Gene symbol	log2FoldChange	*P*-adjusted	Gene Biotype	Description
ENSG00000107018	RLN1	-47.498	0.0001332	Protein coding	Relaxin 1
ENSG00000112414	ADGRG6	18.583	0.0001334	Protein coding	Adhesion G protein-coupled receptor G6
ENSG00000013588	GPRC5A	16.783	0.0002031	Protein coding	G protein-coupled receptor class C group 5 member A
ENSG00000287063		-23.868.	0.0006016	lncRNA	Novel transcript
ENSG00000260213	CENPN-AS1	-20.641	0.003322	Protein coding	Centromere protein N antisense RNA 1
ENSG00000176204	LRRTM4	-37.155	0.003967	Protein coding	Leucine-rich repeat transmembrane neuronal 4
ENSG00000166825	ANPEP	-28.043	0.011940	Protein coding	Aminopeptidase N
ENSG00000260947		-17.482	0.011940	lncRNA	Novel transcript
ENSG00000285741		-42.320	0.011940	lncRNA	Novel transcript
ENSG00000135747	ZNF670	15.990	0.014072	Protein coding	Zinc finger protein 670 to zinc finger protein 695
ENSG00000214195	HMGN2P31	-50.976	0.018926	Protein coding	High mobility group nucleosome-binding domain 2 pseudogene 31
ENSG00000115361	ACADL	-15.392	0.022160	Protein coding	Acyl-CoA dehydrogenase long chain
ENSG00000166451	CENPN	-16.821	0.023411	Protein coding	Centromere protein N
ENSG00000198691	ABCA4	14.148	0.028175	Protein coding	ATP binding cassette subfamily A member 4
ENSG00000188505	NCCRP1	19.607	0.031896	Protein coding	Nuclear and cytoplasmic compartment signaling related protein 1
ENSG00000082196	C1QTNF3	12.687	0.044220	Protein coding	C1q and tumor necrosis factor related protein 3
ENSG00000149636	DSN1	1.775	0.057058	Protein coding	MIS12 kinetochore complex component
ENSG00000089685	BIRC5	12.733	0.059458	Protein coding	Baculoviral IAP repeat containing 5
ENSG00000097046	CDC7	11.668	0.059458	Protein coding	Cell division cycle 7
ENSG00000143320	CRABP2	16.773	0.059458	Protein coding	Cellular retinoic acid binding protein 2
ENSG00000145386	CCNA2	12.156	0.059458	Protein coding	Cyclin A2
ENSG00000164638	SLC29A4	11.797	0.059458	Protein coding	Solute carrier family 29 member 4
ENSG00000175063	UBE2C	13.464	0.059458	Protein coding	Ubiquitin conjugating enzyme E2 C
ENSG00000189056	RELN	-19.215	0.059458	Protein coding	Reelin
ENSG00000196358	NTNG2	-15.599	0.059458	Protein coding	Netrin G2
ENSG00000225885		11.488	0.059458	lncRNA	Novel transcript
ENSG00000230190		-34.930	0.059458	lncRNA	Novel transcript
ENSG00000232855		-11.790	0.059458	lncRNA	Novel transcript
ENSG00000263639	MSMB	-23.403	0.059458	Protein coding	Microseminoprotein beta
ENSG00000274127		-32.485	0.059458	lncRNA	Novel transcript
ENSG00000229980	TOB1-AS1	-0.9964	0.059636	Protein coding	TOB1 antisense RNA 1
ENSG00000235140		-19.616	0.060236	lncRNA	Novel transcript
ENSG00000237492	OR2L9P	-15.926	0.060236	Protein coding	Olfactory receptor family 2 subfamily L member 9 pseudogene
ENSG00000076003	MCM6	1.8987	0.067290	Protein coding	Minichromosome maintenance complex component 6
ENSG00000159708	LRRC36	16.573	0.06908	Protein coding	Leucine rich repeat containing 36
ENSG00000229178		-17.915	0.06908	lncRNA	Novel transcript
ENSG00000171862	PTEN	-1.4253	0.06908	Protein coding	Phosphatase and tensin homolog
ENSG00000272622		-14.766	0.07307	lncRNA	Novel transcript
ENSG00000239946	ZBTB20-AS3	-11.712	0.07507	Protein coding	ZBTB20 antisense RNA 3
ENSG00000260719		-16.982	0.07939	lncRNA	Novel transcript
ENSG00000166803	PCLAF	11.417	0.08201	Protein coding	PCNA clamp associated factor
ENSG00000120903	CHRNA2	-15.444	0.08363	Protein coding	Cholinergic receptor nicotinic alpha 2 subunit
ENSG00000261379		-19.556	0.08363	lncRNA	Novel transcript
ENSG00000104368	PLAT	11.103	0.08784	Protein coding	Plasminogen activator, tissue type
ENSG00000138061	CYP1B1	12.275	0.08784	Protein coding	Cytochrome P450 family 1 subfamily B member 1
ENSG00000166105	GLB1L3	-16.655	0.08784	Protein coding	Galactosidase beta 1 like 3
ENSG00000159713	TPPP3	13.776	0.08927	Protein coding	Tubulin polymerization promoting protein family member 3
ENSG00000101003	GINS1	11.686	0.08940	Protein coding	GINS complex subunit 1
ENSG00000236466	RCAN2-DT	-13.122	0.09024	Protein coding	RCAN2 divergent transcript

ENSG00000158089	GALNT14	16.454	0.09192	Protein coding	Polypeptide N-acetylgalactosaminyltransferase 14
ENSG00000187080	OR2AK2	-21.348	0.09192	Protein coding	Olfactory receptor family 2 subfamily AK member
ENSG00000278683		-24.139	0.09192	lncRNA	Novel transcript
ENSG00000189229		19.762	0.09333	lncRNA	Novel transcript
ENSG00000187957	DNER	-23.747	0.09776	Protein coding	Delta/notch like EGF repeat containing
ENSG00000137142	IGFBPL1	13.691	0.09847	Protein coding	Insulin like growth factor binding protein like 1
ENSG00000236857	RAP1BP1	-24.714	0.09847	Protein coding	RAP1B, member of RAS oncogene family binding protein 1
ENSG00000258038	LINC02327	-36.466	0.09847	lncRNA	Long intergenic non-protein coding RNA 2327
ENSG00000274118		-31.104	0.09847	lncRNA	Novel transcript
ENSG00000117724	CENPF	10.570	0.09857	Protein coding	Centromere protein F
ENSG00000175325	PROP1	-14.350	0.09857	Protein coding	Prop paired-like homeobox 1
ENSG00000257242	LINC01619	-14.748	0.09857	lncRNA	Long intergenic non-protein coding RNA 1619
ENSG00000267218		-20.117	0.09857	lncRNA	Novel transcript

Table S6: nCounter raw counts data for *PTEN*, *EZH2*, housekeeping genes, negative, and positive controls in patients from ADT cohort.

patient	PTEN	EZH2	ACTB	GAPDH	GUSB	HPRT1	RPL13A	NEG_A	NEG_B	NEG_C	NEG_D	NEG_E	NEG_F	POS_A	POS_B	POS_C	POS_D	POS_E	POS_F
**P_001**	1274	98	10369	2602	1108	56	5963	2	6	5	3	6	2	87747	10778	3897	644	221	66
**P_002**	5432	143	91276	13026	1435	239	18562	6	4	5	2	8	5	91572	11223	3889	665	244	72
**P_003**	2590	180	38005	6379	2081	198	11491	5	10	3	5	10	4	84901	10572	3859	576	184	73
**P_004**	2959	376	56561	12639	2546	301	16593	5	5	5	2	9	6	80221	9557	3445	552	221	60
**P_005**	1063	55	17175	2792	744	50	8239	1	4	2	3	8	3	102926	12803	4627	687	250	64
**P_006**	1896	117	27753	4286	1700	99	10995	6	7	4	4	5	6	82649	10005	3521	592	196	73
**P_007**	4018	440	78207	23537	2826	608	15950	6	15	9	7	15	7	73570	9116	3149	502	177	59
**P_008**	1542	227	33047	9608	2561	319	40398	11	10	8	7	10	4	78710	9697	3447	581	206	46
**P_009**	2160	274	59324	5835	1024	176	6725	8	15	3	1	11	6	93508	11739	4097	655	244	67
**P_010**	3776	445	64197	32638	1782	391	20643	3	16	9	8	11	9	98264	12160	4276	645	248	82
**P_011**	452	92	16299	1003	284	18	4137	8	20	6	1	10	2	93873	11573	4108	622	245	87
**P_012**	6348	471	119907	29482	2516	654	26147	10	19	8	6	9	12	91606	11328	3994	608	207	75
**P_013**	1612	63	28842	3347	1399	64	7734	7	11	7	2	8	4	84563	10523	3792	558	236	63
**P_014**	2627	205	52287	9158	1724	148	15239	9	6	7	7	6	4	75836	9526	3340	491	183	50
**P_015**	4315	51	49584	6364	1158	132	10026	5	6	9	3	5	9	70372	8427	2904	476	184	57
**P_016**	3445	70	29342	4175	1110	67	15817	11	11	7	3	11	8	79369	9637	3392	538	163	49
**P_017**	1151	72	16380	4531	892	149	6728	5	11	12	4	12	4	81062	9706	3343	518	214	54
**P_018**	2297	294	29213	7614	1826	138	19783	18	26	11	7	13	2	106608	13128	4390	690	273	78
**P_019**	1820	163	31770	4439	1616	109	10723	6	20	13	3	12	7	82217	10166	3471	550	191	71
**P_020**	208	37	1877	656	202	17	2454	11	12	10	8	5	9	93329	11538	3985	615	235	70
**P_021**	2242	263	36615	9129	1826	205	11597	19	19	13	10	13	10	92635	11378	3961	644	256	83
**P_022**	2863	140	32854	5783	1380	84	12253	9	8	8	8	7	9	91861	11146	3908	613	198	82
**P_023**	3723	303	31916	22102	1285	296	63916	9	8	8	4	5	4	100122	12062	3926	680	224	72
**P_024**	707	54	15908	3244	761	57	8909	16	22	19	11	8	9	123221	14935	5171	814	298	104
**P_025**	4022	75	35493	7152	2340	204	7749	1	6	7	3	16	4	87663	10668	3745	577	252	74
**P_026**	1077	30	19540	1355	680	30	5488	2	2	1	3	1	1	64973	7794	2811	456	177	52
**P_027**	1193	82	28580	4834	873	73	9967	4	3	2	3	7	4	72999	8787	3257	491	178	48
**P_028**	4206	139	38509	7396	1888	144	18973	3	2	6	7	14	6	74624	9112	3205	554	201	66
**P_029**	464	51	7354	867	416	18	3368	1	1	3	1	3	1	74060	9022	3162	509	172	65
**P_030**	2879	177	23314	4038	1119	87	7883	1	1	6	2	1	4	84424	10264	3758	537	217	51
**P_031**	3751	288	60937	20461	3340	547	15057	7	14	7	5	8	3	82492	10085	3603	572	211	86
**P_032**	1589	55	13464	2460	916	44	7823	2	6	4	1	5	4	85003	10276	3700	590	201	44
**P_033**	2256	290	60399	17075	2015	435	16781	6	5	8	6	8	7	76870	9338	3298	530	188	68
**P_034**	588	67	17153	5939	606	177	9097	3	3	7	3	4	1	82966	9920	3567	556	234	52
**P_035**	1846	970	75020	25513	3177	883	24318	2	5	7	3	2	9	66157	8221	2859	493	160	50
**P_036**	1035	120	20049	4485	604	148	7419	3	3	2	1	3	1	81270	9868	3447	562	207	67
**P_037**	1507	60	13955	2001	719	72	5081	5	2	8	5	5	5	83982	10273	3453	547	212	59
**P_038**	2447	196	42761	11961	2205	230	14452	4	2	11	4	6	6	71663	8511	2984	504	173	59
**P_039**	3455	141	27293	5112	1959	133	12942	4	7	7	4	4	1	76309	9281	3290	512	189	63
**P_040**	3571	666	36415	5859	3481	268	24942	5	4	6	4	9	4	66109	8141	2877	494	166	62
**P_041**	1394	91	28155	5037	869	149	7630	9	4	7	3	8	3	77965	9646	3351	535	197	70
**P_042**	1962	109	21004	4627	1214	111	13768	4	4	10	5	10	4	68857	8312	2950	463	152	52
**P_043**	4453	870	125359	20414	4170	751	11359	9	5	8	7	16	5	70541	8461	2851	489	197	60
**P_044**	2583	269	47792	12037	2247	346	13177	5	3	11	8	11	1	77025	9349	3273	520	186	62

**P_045**	1277	31	19853	2394	557	54	6659	4	5	12	2	9	2	82130	10023	3617	540	196	70
**P_046**	10085	405	103921	22645	2328	620	12599	6	8	11	1	8	7	69597	8338	2883	451	171	56
**P_047**	5129	541	56401	14531	3958	358	17621	10	6	9	4	8	6	78011	9472	3158	618	189	61
**P_048**	2751	148	51727	11419	4085	346	14844	3	5	16	6	12	7	104690	11941	5187	626	296	96
**P_049**	4091	562	89100	24392	4130	476	17600	3	5	7	4	15	2	85373	9706	4427	455	228	67
**P_050**	2572	53	14218	6825	965	137	7123	8	10	11	5	14	5	122878	13614	6058	741	322	104
**P_051**	2044	136	22866	3582	1383	121	11365	12	9	19	5	15	10	125640	14218	6073	745	314	77
**P_052**	5483	170	44489	12136	3018	244	28430	6	9	12	1	8	7	108867	12522	5627	666	318	89
**P_053**	6761	600	48098	32654	4806	737	60222	8	6	11	2	14	3	92612	10488	4647	557	267	85
**P_054**	12738	76	118385	16541	2326	433	17552	8	5	11	2	7	6	91814	10369	4656	541	234	78
**P_055**	8177	412	110200	20650	3501	1075	19615	6	7	10	2	11	9	103704	11803	5250	622	289	76
**P_056**	7404	148	88612	19440	2648	402	21010	9	11	10	11	10	8	112248	12671	5601	659	295	91
**P_057**	10686	421	99907	42845	6931	1839	34412	10	5	9	2	18	4	90140	10358	4654	555	247	90
**P_058**	2907	57	31704	5025	928	114	8047	8	2	7	1	8	4	58780	6002	2972	340	173	32
**P_059**	6493	936	77450	34645	6153	1058	21905	8	9	18	4	18	7	110884	12671	5637	690	306	97
**P_060**	3085	793	43178	11054	3153	537	22024	14	6	9	6	7	2	100585	11495	5107	581	297	86
**P_061**	5556	310	24717	8954	3229	194	30833	8	14	13	5	13	8	117812	13212	5972	687	311	95
**P_062**	7040	150	55475	14259	3577	429	31045	9	11	8	8	14	4	100945	11590	5268	615	276	89
**P_063**	6514	579	60892	14478	5161	703	38062	17	6	7	4	13	9	101205	11820	5202	622	269	77
**P_064**	4965	391	96824	18278	4711	944	27316	9	6	5	4	20	8	103719	12004	5489	621	293	73
**P_065**	5467	78	83005	10609	1860	285	12277	4	3	12	4	8	3	112884	12972	5818	703	326	85
**P_066**	4365	53	27068	3896	1348	92	10066	11	3	3	3	12	4	121125	13960	6347	687	312	79
**P_067**	6085	133	48443	8499	2255	269	18940	10	8	5	4	20	4	101411	11805	5440	599	269	73
**P_068**	7054	986	31346	35693	4240	1386	34159	11	7	10	4	11	6	100715	11583	5229	569	261	82
**P_069**	626	99	9757	1527	695	28	4365	15	4	8	4	10	5	121925	14150	6435	688	373	88
**P_070**	3663	257	49020	22954	2579	370	17667	11	5	8	1	9	5	105715	12003	5590	596	285	69
**P_071**	3937	208	34951	6333	1354	119	23934	9	4	9	5	13	6	116002	13659	6092	704	301	95
**P_072**	7351	1053	142237	31784	4073	1668	27638	6	7	14	3	18	8	100612	12007	5297	601	288	81
**P_073**	3412	199	19379	4531	1108	387	23692	15	9	8	5	19	7	121620	14011	6258	755	286	95
**P_074**	3525	485	94161	26077	4780	737	61974	10	6	10	4	11	5	86402	9999	4306	500	259	72
**P_075**	7491	451	99875	27326	5303	780	24520	7	5	8	1	13	8	94458	10652	4895	580	274	74
**P_076**	6055	330	48779	21022	5423	791	8698	10	5	6	5	13	3	111629	12601	5591	650	307	77
**P_077**	3920	484	71748	10498	3521	414	13712	9	2	3	1	16	2	109320	12329	5426	621	294	116
**P_078**	3938	320	53681	13282	3004	374	25766	9	3	10	3	13	3	102764	11934	5327	647	288	66
**P_079**	4819	204	31737	11115	1578	331	25633	8	3	9	3	5	7	98106	11094	5064	549	291	78
**P_080**	9075	395	79201	22837	3605	704	35862	10	6	9	1	14	5	89603	10260	4458	590	240	86
**P_081**	1789	728	74743	34330	2591	1069	38304	10	8	4	7	17	3	113564	12964	5692	673	324	90
**P_082**	5240	180	62817	10854	3170	328	21519	11	3	3	4	10	2	108061	12128	5389	611	290	71
**P_083**	5846	197	83131	23622	5105	537	25247	12	2	8	3	16	6	94003	10603	4682	566	259	79
**P_084**	3480	104	23112	5279	2303	136	13920	9	2	8	2	13	2	93947	10754	4805	576	263	80
**P_085**	3688	197	22168	9339	2272	156	21007	8	4	11	9	10	4	103314	11963	5392	602	262	95
**P_086**	1389	17	20205	2754	306	55	4530	3	1	3	1	7	5	88819	9933	4840	538	238	60
**P_087**	1428	21	12271	1368	320	34	3005	2	1	4	1	2	1	76176	8234	4156	419	192	41
**P_088**	1060	17	11967	1154	342	41	1460	1	2	3	1	5	1	77976	8740	4299	478	220	56
**P_089**	5136	101	40244	10008	1367	251	14857	4	3	2	3	9	1	69408	7549	3655	370	181	50
**P_090**	4594	34	38151	7850	1429	213	9607	2	1	1	1	2	3	56290	6319	2931	340	160	47
**P_091**	3047	172	54097	9044	2513	325	11276	1	7	3	1	9	1	77841	8645	4200	511	214	50
**P_092**	2815	603	50879	14242	4874	580	12030	2	5	3	3	3	1	82099	8792	4351	465	228	60

**P_093**	1177	32	25116	4052	460	51	7773	4	1	4	1	4	5	58818	6100	3114	367	172	45

Table S7: nCounter raw counts data for *PTEN*, *EZH2*, housekeeping genes, negative, and positive controls in patients from ADT+D cohort.

patient	**PTEN**	**EZH2**	**ACTB**	**GAPDH**	**GUSB**	**HPRT1**	**RPL13A**	**NEG_A**	**NEG_B**	**NEG_C**	**NEG_D**	**NEG_E**	**NEG_F**	**POS_A**	**POS_B**	**POS_C**	**POS_D**	**POS_E**	**POS_F**
**S_001**	7735	477	42230	17115	3434	945	14977	10	10	7	2	16	8	93129	10638	4740	585	240	100
**S_002**	8728	509	133841	33376	13459	2254	34008	11	5	7	2	16	10	79647	9093	4082	511	231	68
**S_003**	2862	56	26689	4052	1101	84	8305	11	12	17	3	12	7	128121	14962	6636	766	366	91
**S_004**	1682	139	67262	8841	1187	123	14401	11	9	13	3	15	10	135031	15241	6818	836	352	97
**S_005**	356	48	28831	10392	392	152	9423	10	12	17	8	13	8	131767	14921	6660	739	354	94
**S_006**	8497	370	175653	33669	7045	994	28148	6	4	6	4	14	8	81851	9536	4138	496	216	62
**S_007**	4778	210	43930	8445	2428	303	16203	7	5	14	6	15	5	105522	11692	5595	618	277	91
**S_008**	11788	253	78977	25215	4466	707	34994	14	8	20	2	15	11	74185	8188	3864	436	215	78
**S_009**	9261	616	89867	30085	5771	783	19605	7	5	8	7	14	5	88270	9506	4647	538	229	67
**S_010**	10792	380	89274	40197	4039	1115	37299	9	4	12	7	17	5	70997	7784	3747	443	201	57
**S_011**	9242	590	120353	32916	6118	1156	24174	13	3	15	3	21	3	82862	8945	4261	451	252	67
**S_012**	12220	486	95076	29173	6375	1016	36978	13	10	12	5	14	7	88765	10211	4559	542	275	83
**S_013**	5545	223	83857	22008	3485	681	18261	4	11	12	6	20	7	93824	10700	4991	626	226	64
**S_014**	15104	247	133525	22525	3547	820	32637	19	6	8	6	15	7	95684	10841	5045	577	291	95
**S_015**	8699	809	194297	31819	6323	996	23543	15	5	14	1	30	2	80677	9043	4269	506	244	62
**S_016**	2077	130	36406	4887	1531	148	14907	14	5	8	5	14	2	114434	12936	6019	651	317	74
**S_017**	2771	59	26121	3994	1362	70	10201	7	5	9	3	8	5	91409	10217	4795	569	242	86
**S_018**	6793	154	67472	12568	1577	354	11368	6	2	5	2	10	6	71312	7762	3704	417	193	58
**S_019**	4157	423	67884	10811	1928	213	11276	4	6	12	1	13	5	96204	10693	5034	567	254	64
**S_020**	2483	143	37999	11195	2047	196	17773	7	12	7	4	10	4	75805	8388	4023	433	215	48
**S_021**	3979	312	27528	7912	2551	185	21104	5	3	11	4	13	5	78276	8822	4453	420	224	46
**S_022**	5718	351	48122	15636	3263	375	16523	10	5	4	3	6	6	82627	8757	4215	504	232	75
**S_023**	9353	278	106956	29627	3854	940	24433	5	12	11	5	21	3	70449	7858	3635	450	232	88
**S_024**	12327	496	88993	31217	4303	1045	26225	16	6	9	2	25	6	66188	7418	3574	407	189	75
**S_025**	2607	168	41327	11537	3866	208	17575	14	3	8	3	15	6	86935	9446	4565	516	257	85
**S_026**	5743	117	59755	9297	2097	245	12738	12	8	9	4	13	4	77571	8558	4080	485	200	64
**S_027**	5048	275	45401	13047	2571	281	23367	9	8	13	5	14	6	86968	9886	4636	550	257	88
**S_028**	1313	46	11660	2999	569	114	10150	3	2	4	3	4	2	100744	11122	5482	590	256	69
**S_029**	6277	376	54276	8114	3680	323	9971	7	4	3	5	7	4	84301	9135	4448	507	238	68
**S_030**	3244	159	48832	9612	2965	552	3907	5	3	11	2	8	5	84042	9357	4447	490	231	53
**S_031**	8794	229	96160	25935	2972	733	22924	2	5	7	1	12	10	84817	9203	4445	488	241	69
**S_032**	2567	132	32904	9395	3866	350	8660	2	1	5	2	4	1	74258	8469	3908	434	191	51
**S_033**	3871	253	71637	13857	1483	409	15035	6	3	3	4	9	8	54835	5658	4633	356	113	53
**S_034**	4708	205	90327	16426	1888	575	10943	7	4	10	6	8	4	62406	6349	5127	433	113	46
**S_035**	518	44	11739	4053	371	91	8195	3	4	8	2	6	1	66772	6948	5649	404	156	52
**S_036**	3212	295	63320	12003	1469	467	9285	5	6	5	4	8	7	58548	5932	4903	365	137	45
**S_037**	3632	95	46065	13317	1356	398	15510	2	5	8	4	7	1	61329	6303	5134	395	135	44
**S_038**	6888	188	47212	19300	1990	559	30669	9	7	9	4	5	9	64498	6669	5416	431	154	61
**S_039**	4638	398	100766	29268	2876	677	12463	8	5	4	7	17	14	82306	8694	6706	496	173	66
**S_040**	3116	371	30439	7300	1701	331	18132	2	5	6	4	11	8	71107	7628	5903	454	146	45
**S_041**	4913	196	87295	14446	2007	358	8671	7	9	10	5	13	7	79345	8427	6729	493	179	59
**S_042**	6243	123	45286	13206	2223	410	13487	11	4	13	6	6	5	66747	7002	5441	436	136	60
**S_043**	2476	117	51398	8709	1738	315	15259	7	5	14	7	16	4	80180	8563	6661	503	183	50
**S_044**	3064	1233	70017	41400	5675	594	8502	14	13	10	10	36	8	104848	11112	8326	668	233	80

**S_045**	1362	118	8169	5584	823	127	11929	10	11	9	6	10	7	104757	10998	8437	697	250	84
**S_046**	2990	156	30059	6008	1615	212	15071	9	9	14	5	13	13	96054	10015	7715	590	253	77
**S_047**	3973	284	25644	11851	2121	332	22567	11	12	14	5	10	6	84906	8803	6526	547	206	62
**S_048**	2881	180	26675	7328	1373	159	20519	13	8	5	5	11	9	101481	10528	8171	674	217	84
**S_049**	3758	107	24970	7340	1242	171	21965	5	10	12	4	13	7	97360	10153	7686	596	208	75
**S_050**	3967	116	35643	7653	1322	339	18575	13	14	11	5	12	4	81598	8686	6438	527	195	71
**S_051**	1468	135	26761	7449	1005	182	7945	5	13	5	2	10	10	95402	10202	7550	624	227	60
**S_052**	4682	119	44944	6802	1215	265	11506	7	9	17	2	15	5	90818	9757	7221	563	227	83
**S_053**	1131	63	27896	5787	319	124	4391	1	2	8	3	11	6	91162	9595	7264	554	195	61
**S_054**	1618	92	21701	4616	581	83	9385	10	9	14	4	12	13	102873	10947	8120	625	204	91
**S_055**	2751	432	37942	11160	1341	171	10244	11	7	11	5	10	11	108127	11559	8503	714	256	80
**S_056**	3548	131	35198	9562	2114	245	18778	9	11	13	8	15	8	112141	11610	9051	657	218	76
**S_057**	2290	345	27463	5415	1404	191	11133	10	5	15	5	16	14	128104	13170	10020	790	286	100
**S_058**	3251	166	65380	19534	1744	414	34940	15	4	7	8	17	3	104337	10833	8451	646	234	91
**S_059**	4819	261	55750	23970	2930	679	26355	16	16	22	6	12	12	109463	11421	9002	667	241	66
**S_060**	8117	570	47305	16923	5211	438	24573	13	7	11	7	20	23	90547	9153	7485	631	258	83
**S_061**	1073	341	29047	12117	2043	322	9797	5	5	11	7	18	2	96988	9727	7815	638	242	77
**S_062**	5316	194	37195	10460	1266	674	34447	12	14	9	7	18	10	100915	10428	8156	683	231	87
**S_063**	1334	50	16289	5764	783	135	9159	7	14	14	4	4	3	104661	10823	8418	675	232	67
**S_064**	1936	309	45053	15133	2449	368	18600	14	8	16	7	14	11	104667	11060	8360	622	248	93
**S_065**	5268	296	34906	8143	2287	257	27964	12	7	15	9	19	12	100616	10841	8096	696	250	67
**S_066**	6811	271	57167	17454	2231	362	18021	11	10	19	4	10	12	110951	11851	9128	731	249	86
**S_067**	3940	93	70057	20843	3981	664	27775	15	8	16	8	22	6	107911	11432	8611	702	244	86
**S_068**	4025	177	41999	14215	1747	400	20779	12	11	12	2	21	12	92434	9610	7331	603	222	79
**S_069**	8998	221	65890	10149	2475	396	16272	8	6	5	5	19	14	101424	10363	8118	627	208	62
**S_070**	2161	919	81793	37845	2752	880	8951	10	7	12	3	11	11	119680	12572	9429	742	275	91
**S_071**	11054	220	52542	20661	3056	741	20631	14	12	10	5	22	11	77899	8228	6271	516	214	77
**S_072**	5373	325	95208	18802	3121	695	30104	9	6	13	4	15	13	77350	8009	6189	468	199	60
**S_073**	5862	418	51866	9460	2199	322	21420	11	6	15	5	14	7	100689	10421	8110	649	241	81
**S_074**	5117	328	37409	16701	2188	419	31514	12	12	9	5	20	17	93620	9949	7690	638	225	90
**S_075**	27481	3544	145013	31086	6822	2146	10572	11	9	18	4	24	17	92492	9805	7607	575	207	74
**S_076**	2390	142	27691	7894	1448	124	9588	3	11	6	4	7	6	111009	11583	8775	688	260	83
**S_077**	5718	470	88961	39063	4478	790	20911	9	10	16	10	28	5	94693	9931	7869	592	201	62
**S_078**	2253	123	24215	4223	1355	141	10750	4	6	13	8	10	11	106364	11147	8617	682	233	72
**S_079**	1889	276	12081	2933	1843	58	6334	11	8	12	3	16	8	102873	10770	8417	664	230	80
**S_080**	3380	94	14132	10558	1225	355	7999	8	7	7	3	10	12	92748	9640	7512	546	216	57
**S_081**	2451	71	28332	4979	962	123	8833	7	8	12	3	15	6	109718	11472	8854	689	228	87
**S_082**	2817	1188	53414	48559	5196	849	53031	11	11	11	8	13	11	101535	10692	8188	639	268	77
**S_083**	4000	219	28804	50138	957	349	44002	17	13	7	9	8	9	109984	11700	8957	697	247	105
**S_084**	1007	47	10575	3980	250	80	6995	5	10	12	8	10	9	112622	11986	9149	717	255	75
**S_085**	323	50	3609	1842	224	51	5664	7	3	7	6	9	5	113014	11985	9133	678	243	71
**S_086**	966	71	8794	1810	461	63	3905	11	6	13	6	11	7	130204	13762	10473	851	309	93
**S_087**	2771	103	25781	7657	1674	257	14224	10	5	20	5	17	6	122176	12943	9886	797	309	120
**S_088**	635	37	6809	2578	293	51	6078	8	8	9	8	17	8	145331	15006	11751	984	347	112
**S_089**	121	64	7508	1787	169	111	3543	1	2	4	2	7	2	111345	11772	8721	710	260	75
**S_090**	5730	77	20303	3715	1071	150	7339	3	8	6	7	16	13	117921	12335	9486	729	258	87
**S_091**	1319	92	8374	2595	1131	71	6489	6	6	8	3	7	3	105938	11118	8577	657	223	78
**S_092**	2632	40	11479	2055	823	50	6256	5	5	8	2	12	5	125605	13354	10151	809	286	107

**S_093**	3078	188	51048	19130	1165	593	29659	12	7	12	7	16	11	115395	12407	9462	754	266	80
**S_094**	800	39	16279	3155	317	83	4686	4	11	5	3	8	10	117106	12533	9271	705	278	85
**S_095**	3053	140	31642	10602	1082	254	22369	8	7	14	1	16	8	106319	11312	8434	666	237	90
**S_096**	702	23	9730	1958	196	38	7280	8	6	10	1	10	12	123746	13210	10176	799	294	94
**S_097**	834	19	10351	1283	315	38	2691	5	5	13	4	6	7	118311	12599	9686	720	259	84
**S_098**	893	126	22349	4934	1101	111	8193	6	8	7	6	10	8	109835	11627	8745	684	260	74
**S_099**	1298	90	13015	3321	708	92	7582	5	8	9	6	8	8	123376	12903	9897	794	254	91
**S_100**	992	59	24556	2260	519	84	3976	11	11	10	7	8	12	123254	12964	10002	751	252	78
**S_101**	1781	24	20608	2652	419	100	4951	7	8	8	4	17	12	123107	12766	9769	802	273	94
**S_102**	726	58	19155	6517	299	95	2171	5	7	11	2	10	7	122351	12886	9643	803	293	91
**S_103**	4971	587	40619	23322	2720	670	18683	17	9	11	7	20	10	53329	5748	4480	341	147	60
**S_104**	8360	664	48061	33977	3282	765	14559	10	23	17	9	10	15	52465	5754	4529	353	126	69
**S_105**	6125	246	47442	10792	2410	627	28536	8	10	14	8	18	7	67177	7112	5727	447	148	73
**S_106**	6295	403	66357	29720	3401	891	33746	16	21	11	7	22	15	98479	10325	8139	652	217	93
**S_107**	7894	227	46786	9279	2102	426	33814	9	9	12	7	14	14	44787	4805	3792	278	110	40
**S_108**	6121	337	79162	18106	1408	538	24354	10	13	21	7	14	15	118585	12112	9564	788	260	87
**S_109**	1560	528	56134	29978	4216	725	41341	12	19	15	11	36	9	92130	9621	7617	570	208	74
**S_110**	4300	524	65241	16136	3488	627	23191	14	8	20	9	24	7	110187	11764	9194	714	275	104
**S_111**	2661	156	43992	12330	3801	315	24250	17	11	21	12	23	17	119787	12565	9636	805	280	100
**S_112**	5470	255	42838	15722	2588	531	31512	20	29	25	8	20	18	123570	12943	10087	849	284	93
**S_113**	3429	149	47938	20715	1814	611	24239	15	11	17	10	13	17	108729	11309	8609	674	268	100
**S_114**	5120	385	58224	12800	14928	232	18732	10	14	18	8	21	9	99308	10302	8234	657	207	82
**S_115**	2603	72	45905	8972	857	183	7693	4	14	24	9	13	12	152690	15555	12144	1020	326	118
**S_116**	5048	517	58789	31972	3648	1117	21998	12	16	16	4	13	2	116289	11915	9434	778	257	117
**S_117**	6647	322	70415	19946	3286	433	17517	13	15	26	14	19	16	115928	11832	9350	723	247	80
**S_118**	5872	216	58110	12072	2200	230	11861	13	9	16	13	26	4	115984	12331	9306	724	239	87
**S_119**	7031	309	57268	10985	8161	672	40848	13	15	26	7	12	15	123680	12653	9972	841	267	99
**S_120**	6288	472	41640	42522	3471	615	29013	7	14	18	3	9	14	102695	10706	8114	641	228	86
**S_121**	6036	401	50746	18245	2217	359	21191	9	18	25	13	27	11	108406	11169	8528	768	236	99
**S_122**	2506	397	67930	13749	2239	425	24035	17	18	7	5	8	10	89904	9329	7158	586	212	80
**S_123**	2937	129	53539	12530	1514	293	12848	10	10	11	9	26	2	99445	10630	7844	641	229	79
**S_124**	618	46	6850	1212	507	25	4051	13	9	17	5	14	8	38101	3937	3254	257	93	47
**S_125**	3928	94	40353	6436	1104	308	10977	10	6	15	3	25	6	110642	11159	8638	731	264	81

Table S8: nCounter raw counts data for *PTEN*, *EZH2*, housekeeping genes, negative, and positive controls in patients from ADT+ARSI cohort.

patient	PTEN	EZH2	ACTB	GAPDH	GUSB	HPRT1	RPL13A	NEG_A	NEG_B	NEG_C	NEG_D	NEG_E	NEG_F	POS_A	POS_B	POS_C	POS_D	POS_E	POS_F
**N_001**	15787	243	152386	30903	4213	931	20083	13	7	10	1	22	5	77236	8379	3941	488	222	69
**N_002**	8143	530	122253	30593	4646	805	36123	16	11	21	6	42	23	78242	8393	6510	482	188	73
**N_003**	11842	666	115792	42107	4320	1479	70667	16	14	19	8	40	11	59623	6563	5084	387	152	62
**N_004**	23798	367	235364	41234	7998	1527	53316	8	10	18	7	30	14	83633	9002	6999	521	216	66
**N_005**	8511	142	54445	19257	2100	385	40218	10	7	15	7	18	17	92320	10114	7715	606	236	88
**N_006**	3891	213	44479	5394	2069	128	25022	4	9	15	2	18	17	119792	12694	10049	698	277	79
**N_007**	6292	270	51800	11514	2826	562	15702	18	8	24	16	30	18	58763	6367	5037	446	183	83
**N_008**	8544	81	82999	15313	2085	509	18104	13	9	19	9	38	26	75245	8235	6472	556	208	101
**N_009**	7542	236	54284	11824	2667	463	19556	15	23	24	2	44	18	57813	6631	5207	470	179	91
**N_010**	2148	143	27232	3545	1324	133	7241	9	10	16	8	16	19	75718	8225	6461	582	208	99
**N_011**	6009	256	40675	9617	1686	440	18990	20	13	17	12	24	12	77232	8541	6781	556	214	92
**N_012**	1669	152	40162	7363	1387	234	18891	17	9	19	6	25	26	61150	7061	5409	474	178	91
**N_013**	5121	202	52285	11394	1780	368	19699	16	20	14	5	50	21	52208	6052	4752	376	149	79
**N_014**	2389	110	39567	10334	1520	426	8645	9	13	13	6	30	20	60656	7186	5412	474	162	60
**N_015**	2337	73	20664	4181	1117	134	8858	9	5	7	4	10	7	54404	6337	4859	389	138	66
**N_016**	2038	89	51578	7663	1239	237	9631	6	5	9	5	11	13	53776	6408	4947	340	145	57
**N_017**	2253	408	46929	11710	3039	387	6097	9	9	9	6	15	12	54279	6250	5063	432	162	76
**N_018**	4973	78	64232	9462	1899	378	14918	18	10	13	3	30	20	58081	6550	5112	403	156	63
**N_019**	785	35	8563	1021	543	33	4453	4	6	6	5	5	3	54294	5915	4639	389	161	49
**N_020**	443	56	7211	1355	971	28	5735	1	3	8	2	5	4	56697	6310	5045	379	136	53
**N_021**	1239	64	11674	4289	306	130	5041	4	8	3	4	7	2	49724	5603	4598	373	139	44
**N_022**	982	135	16799	1682	1094	77	2830	7	2	12	5	7	1	58380	6480	5333	447	126	50
**N_023**	3964	175	71948	23853	1662	598	7092	10	14	22	12	23	18	57738	6457	5090	416	170	55
**N_024**	2855	393	43824	6706	1813	814	3944	10	17	18	4	30	22	61351	7068	5494	478	162	85
**N_025**	1433	311	39979	16900	1674	506	17154	10	5	10	4	23	15	30319	3453	2724	227	107	44
**N_026**	729	61	14833	2963	1023	61	7039	5	2	3	1	6	7	30997	3409	2804	246	83	30
**N_027**	2555	96	21316	5289	914	322	7801	8	1	3	1	11	9	40086	4664	3731	265	113	32
**N_028**	2740	83	34285	6005	1230	156	7541	3	3	9	4	17	8	33398	3794	3103	243	96	34
**N_029**	1542	166	25869	7500	976	284	5438	11	5	5	8	14	12	28753	3283	2704	220	90	51
**N_030**	2739	82	28758	6490	1246	206	8923	8	3	3	6	10	10	30909	3623	2894	230	89	48
**N_031**	2500	241	20628	7136	2531	287	10178	13	14	5	2	18	15	34535	3984	3257	297	96	33
**N_032**	1720	194	23231	9806	1990	198	9511	10	4	4	3	12	8	30198	3375	2764	222	86	41
**N_033**	519	90	12234	3928	285	54	12937	4	6	5	2	7	7	35025	3901	3229	238	105	32
**N_034**	2525	60	24375	7665	693	183	8648	2	3	11	2	16	7	31417	3525	2841	222	75	28
**N_035**	1218	12	2117	318	199	8	1440	2	1	1	2	2	1	25667	2910	2330	193	67	28
**N_036**	1915	88	18162	4388	1381	153	9492	6	5	11	4	14	8	33857	3688	3114	277	87	60
**N_037**	2438	151	25088	7017	1091	183	7239	9	9	4	2	16	6	42332	4826	3926	290	119	48
**N_038**	2048	90	16016	4533	692	131	6432	7	4	5	1	11	5	51473	5945	4757	391	129	37
**N_039**	3614	208	30212	9109	4057	237	16721	12	11	7	3	17	13	50425	5901	4803	366	146	60
**N_040**	3644	94	30080	6349	1534	224	10910	9	5	10	6	17	8	54186	6203	5007	394	144	59
**N_041**	3367	134	32744	7734	1718	206	9886	10	5	5	1	17	12	49057	5559	4570	350	128	69
**N_042**	1200	17	13152	1151	140	28	1359	2	1	2	1	8	3	48032	5566	4544	324	128	42
**N_043**	2691	101	42062	7475	1169	324	9390	10	13	10	4	36	18	48386	5576	4525	385	166	42
**N_044**	215	62	12683	3672	437	126	3539	3	3	2	2	6	5	49144	5597	4473	332	115	47

**N_045**	5156	42	29868	6384	1112	238	6710	1	3	9	1	10	7	52961	5947	4980	385	138	40
**N_046**	5927	206	34253	11338	1726	516	9343	15	9	11	3	23	15	40366	4804	4123	327	137	72
**N_047**	416	10	2518	452	123	11	2032	1	1	4	1	5	2	40594	4526	3691	292	97	31
**N_048**	512	33	9446	761	412	10	3254	1	1	5	2	7	1	35674	4011	3372	266	65	33
**N_049**	191	39	3358	568	318	20	1830	1	2	4	1	3	2	42354	4812	3880	294	102	42
**N_050**	387	17	3619	390	419	8	2503	2	2	5	1	3	5	45715	5115	4157	320	115	39
**N_051**	4658	93	31869	10041	5524	301	19648	12	6	9	6	18	9	42393	4722	3904	331	118	46
**N_052**	374	157	17343	14561	858	191	4022	6	7	2	4	18	8	30847	3335	2801	215	89	47
**N_053**	1837	85	24062	5652	578	186	4922	4	1	4	3	8	6	25978	2982	2462	207	73	25
**N_054**	4547	109	40037	5626	1477	238	8431	5	5	15	2	20	6	36775	4027	3312	255	93	31
**N_055**	856	53	13469	2435	408	91	2919	4	3	2	3	2	4	34162	4100	3075	247	96	26
**N_056**	3056	211	20563	14847	1294	323	15952	1	5	5	1	14	14	30174	3268	2766	223	95	42
**N_057**	2584	152	41225	12808	1063	458	7398	8	10	14	2	21	15	33827	3582	3057	259	96	37
**N_058**	3223	34	23008	3797	597	119	7011	5	7	6	4	19	9	29110	3208	2731	197	90	26
**N_059**	2069	99	26142	7821	1123	254	12714	5	6	3	1	10	11	32038	3415	2908	248	91	30
**N_060**	6235	218	40020	8009	1998	236	10787	6	2	3	2	19	18	30506	3225	2738	226	100	39
**N_061**	2554	72	17993	10484	1267	166	10025	8	9	7	4	17	10	25650	2745	2344	182	85	45
**N_062**	1488	78	23805	5017	1157	180	6332	5	6	6	4	18	9	24188	2623	2226	212	76	40
**N_063**	1310	48	14939	2368	992	67	5300	1	4	1	1	4	4	28670	3108	2547	210	79	19
**N_064**	586	29	3349	482	245	11	2453	1	2	3	2	4	3	32085	3635	2965	209	97	37
**N_065**	2152	70	31421	5553	895	165	5353	8	3	5	4	11	9	27439	3034	2415	232	71	35
**N_066**	1752	68	12422	2550	501	85	6186	6	3	3	2	10	5	28967	3186	2691	216	80	44
**N_067**	2017	70	17203	5504	653	130	5925	5	4	2	2	14	5	24947	2734	2229	185	72	36
**N_068**	2392	95	22805	5807	1036	187	11981	7	8	6	3	20	13	25657	2900	2370	214	77	47
**N_069**	1571	21	14746	1892	624	81	3372	2	3	3	2	13	1	23010	2395	2169	187	73	22
**N_070**	1558	48	13572	2662	827	78	4816	3	6	4	2	5	4	24721	2641	2213	193	63	26
**N_071**	2918	81	26837	5483	1120	167	6964	7	9	7	2	9	9	22652	2536	2154	176	73	33
**N_072**	891	19	11218	1486	342	41	2045	2	4	1	1	8	4	20309	2266	1853	167	57	11
**N_073**	724	82	10646	1709	788	43	2845	3	2	1	2	8	5	20464	2201	1960	155	62	28
**N_074**	1910	158	13483	6732	951	189	6429	6	6	7	1	8	4	21227	2357	1941	181	66	29
**N_075**	2306	82	18197	4340	978	197	4296	4	2	5	2	12	7	20758	2317	1961	172	53	35
**N_076**	624	14	2053	375	269	11	1585	3	2	1	1	2	2	32000	3468	2804	266	85	28
**N_077**	278	16	1079	274	116	11	943	2	1	3	1	1	1	26061	2854	2430	183	85	12
**N_078**	1107	721	41729	10621	3306	722	6462	6	6	9	1	17	10	30515	3284	2874	251	79	23
**N_079**	1473	72	27902	4231	844	190	4355	3	3	5	4	13	5	29931	3260	2694	269	85	49
**N_080**	1216	41	10395	2320	545	71	4689	3	1	2	1	6	4	17443	1837	1650	153	48	30
**N_081**	445	20	7173	956	452	32	4101	3	4	5	2	6	2	17370	1857	1659	122	59	22
**N_082**	195	34	4976	960	249	46	1651	4	1	5	1	7	5	17917	1842	1574	137	66	15
**N_083**	270	25	2088	607	169	16	1332	1	1	1	1	1	2	12737	1396	1224	95	33	8
**N_084**	311	37	2904	853	114	24	460	3	1	1	1	2	2	17881	1931	1548	139	48	16
**N_085**	202	25	1891	269	148	5	610	1	1	1	1	3	1	15000	1669	1405	144	37	14
**N_086**	154	29	3157	513	240	17	449	1	1	1	1	2	1	14537	1575	1398	111	39	12
**N_087**	272	15	2095	730	159	12	1968	2	2	3	1	2	1	13327	1429	1231	85	37	19
**N_088**	544	27	7083	2104	381	44	2874	1	1	1	1	6	2	16101	1785	1493	116	48	22
**N_089**	1315	80	12096	2736	1020	85	3420	2	1	3	1	5	7	20174	2218	1914	166	48	20
**N_090**	1046	16	8831	1308	297	37	2580	1	1	1	3	3	6	17402	1982	1595	122	49	20
**N_091**	823	50	9130	2030	999	37	2762	2	2	2	1	4	3	17886	2079	1640	134	44	19
**N_092**	140	65	3366	2812	494	49	4022	2	1	2	1	1	1	17713	1985	1684	136	49	24

**N_093**	2470	79	32913	3340	1369	220	4517	4	4	6	1	10	6	19811	2162	1759	168	52	21
**N_094**	841	79	10976	3436	1000	126	2939	4	1	2	1	8	4	13270	1460	1250	102	35	25
**N_095**	436	63	11447	2593	1112	73	1985	5	7	1	1	4	1	18420	2076	1751	144	49	24
**N_096**	1724	44	15572	4700	2131	117	5879	5	4	1	1	9	9	20618	2285	1818	181	55	20
**N_097**	1436	57	7303	2431	856	56	7408	1	1	1	1	1	1	21426	2356	1967	164	57	15
**N_098**	1569	26	14926	3112	490	130	6973	2	1	1	1	3	3	18967	2096	1725	139	40	13
**N_099**	1073	50	11109	2836	315	100	3786	1	2	1	1	6	5	16315	1754	1485	123	41	15
**N_100**	3444	164	47846	12576	3510	374	17625	15	21	15	4	30	13	44243	5491	4433	413	146	105
**N_101**	1739	40	27426	1249	619	37	3441	5	2	4	3	12	1	62453	7084	5839	514	177	86
**N_102**	1828	112	33731	4768	2893	137	13158	10	13	11	8	22	8	59104	7106	5839	490	200	106
**N_103**	6473	321	66594	21844	2175	423	21168	15	10	19	3	38	16	43306	5031	4100	403	145	96
**N_104**	3084	87	30225	9708	731	232	8542	4	3	8	1	12	6	56609	6684	5414	478	189	54
**N_105**	587	40	23952	3290	438	71	5001	4	1	4	2	9	4	33580	4421	3000	287	98	40
**N_106**	3564	70	49491	8487	1288	281	7584	7	3	4	4	20	15	47267	5884	4599	393	149	52
**N_107**	1786	40	7501	3632	306	66	2246	3	1	6	3	12	5	67219	7457	6016	494	194	88
**N_108**	3291	52	32568	6461	969	192	13856	12	8	4	2	11	8	65939	7258	5989	519	177	65
**N_109**	4374	162	49747	5771	1747	324	7829	11	6	10	3	21	14	64069	7386	5975	505	179	82
**N_110**	1916	87	27121	3494	458	169	11272	9	5	11	3	16	10	53731	6069	4809	438	163	60
**N_111**	234	8	2064	339	174	11	1248	2	1	4	1	3	1	12987	3939	1018	254	73	14
**N_112**	606	40	14237	3474	405	59	6825	2	2	9	1	4	6	59632	14888	4847	1273	318	73
**N_113**	4191	321	57672	9665	2378	288	11472	5	8	3	4	21	10	61065	15565	5156	1309	300	72
**N_114**	6156	211	77460	26143	1805	710	14451	4	8	5	3	20	15	54880	13836	4535	1173	348	77
**N_115**	4643	240	67391	12137	1703	381	17325	9	6	11	3	22	10	54326	14124	4566	1225	320	86
**N_116**	5754	108	65645	9591	1628	308	14585	15	10	5	3	11	14	54063	13895	4400	1208	288	70
**N_117**	7584	269	68971	21401	2639	529	23370	9	10	16	6	22	24	50581	12791	4238	1163	299	86
**N_118**	8141	768	62429	14461	4674	550	15491	14	15	9	8	25	12	72948	18408	5936	1612	419	83
**N_119**	5368	245	57960	18471	2730	584	29433	12	10	9	7	30	13	64234	16342	5406	1392	375	103
**N_120**	13938	298	116701	24608	2873	722	24689	15	13	10	7	29	25	76210	19483	6136	1589	411	117
**N_121**	9928	182	102828	18347	3218	707	16779	10	12	12	6	44	21	67282	16925	5426	1489	399	89
**N_122**	5780	262	79991	15581	3525	509	10837	12	11	7	5	23	19	61698	15550	4930	1308	324	88
**N_123**	5828	228	36789	10888	5803	420	18783	18	8	10	9	27	13	58976	15188	4909	1246	319	100
**N_124**	6757	576	57934	14188	4267	617	17626	12	10	19	10	19	19	71679	18845	5903	1553	413	87
**N_125**	6032	295	42683	18022	4134	392	18060	6	5	8	3	19	15	69633	18045	5672	1488	385	90
**N_126**	5004	218	71524	14583	1224	334	25305	8	14	9	5	24	16	65224	16771	5406	1375	373	86
**N_127**	2837	75	50651	5724	1489	147	9420	8	7	5	4	20	6	69940	18033	5751	1441	369	77
**N_128**	9960	206	69923	20237	3299	671	34556	14	10	10	3	33	22	73148	18572	5854	1520	409	98
**N_129**	6840	239	106088	22161	2354	882	21045	13	12	8	7	30	18	86203	21338	6951	1795	466	109
**N_130**	3084	1046	54948	24093	2368	1254	9972	18	9	7	7	23	8	91384	23033	7317	1937	557	103
**N_131**	10780	413	127053	31707	7643	1039	24541	26	12	16	11	48	27	66592	16990	5443	1479	397	90
**N_132**	12700	296	78309	21928	4757	603	33763	36	12	15	6	34	26	66579	16949	5496	1384	387	103
**N_133**	8473	391	71237	16604	2835	505	18433	7	5	10	4	17	12	60737	13749	4670	1290	396	67
**N_134**	6828	208	81592	16516	3218	570	18281	14	9	7	4	23	18	78971	20149	6335	1662	436	109
**N_135**	2196	148	19425	2458	1191	88	9514	6	8	4	4	12	9	84873	21351	6779	1704	462	109
**N_136**	7511	60	62506	5990	1067	201	16695	5	6	13	3	26	12	86213	22418	6892	1750	516	107
**N_137**	18456	215	111659	17668	6327	632	25592	18	14	8	5	30	20	65237	16763	5410	1404	354	98
